# Activation of Invariant Natural Killer T Cells by α-Galactosylceramide Attenuates the Development of Angiotensin II-Mediated Abdominal Aortic Aneurysm in Obese *ob/ob* Mice

**DOI:** 10.3389/fcvm.2021.659418

**Published:** 2021-05-10

**Authors:** Akimichi Saito, Naoki Ishimori, Satoshi Tokuhara, Tsuneaki Homma, Mikito Nishikawa, Kazuya Iwabuchi, Hiroyuki Tsutsui

**Affiliations:** ^1^Department of Cardiovascular Medicine, Faculty of Medicine and Graduate School of Medicine, Hokkaido University, Sapporo, Japan; ^2^Department of Immunology, Kitasato University School of Medicine, Sagamihara, Japan; ^3^Department of Cardiovascular Medicine, Kyushu University Graduate School of Medicine, Fukuoka, Japan

**Keywords:** obesity, natural killer T cells, mouse, macrophages, inflammation, angiotensin II, aortic aneurysm, α-galactosylceramide

## Abstract

The infiltration and activation of macrophages as well as lymphocytes within the aorta contribute to the pathogenesis of abdominal aortic aneurysm (AAA). Invariant natural killer T (iNKT) cells are unique subset of T lymphocytes and have a crucial role in atherogenesis. However, it remains unclear whether iNKT cells also impact on the development of AAA. *Ob/ob* mice were administered angiotensin II (AngII, 1,000 ng/kg/min) or phosphate-buffered saline (PBS) by osmotic minipumps for 4 weeks and further divided into 2 groups; α-galactosylceramide (αGC; PBS-αGC; *n* = 5 and AngII-αGC; *n* = 12), which specifically activates iNKT cells, and PBS (PBS-PBS; *n* = 10, and AngII-PBS; *n* = 6). Maximal abdominal aortic diameter was comparable between PBS-PBS and PBS-αGC, and was significantly greater in AngII-PBS than in PBS-PBS. This increase was significantly attenuated in AngII-αGC without affecting blood pressure. αGC significantly enhanced iNKT cell infiltration compared to PBS-PBS. The ratio of F4/80-positive macrophages or CD3-positive T lymphocytes area to the lesion area was significantly higher in AngII-PBS than in PBS-PBS, and was significantly decreased in AngII-αGC. Gene expression of M2-macrophage specific markers, arginase-1 and resistin-like molecule alpha, was significantly greater in aortic tissues from AngII-αGC compared to AngII-PBS 1 week after AngII administration, and this increase was diminished at 4 weeks. Activation of iNKT cells by αGC can attenuate AngII-mediated AAA in *ob/ob* mice via inducing anti-inflammatory M2 polarized state. Activation of iNKT cells by the bioactive lipid αGC may be a novel therapeutic target against the development of AAA.

## Introduction

Abdominal aortic aneurysm (AAA) is a common disease that affects 5% of men over the age of 65 years (1). The risk of the rupture of AAA increases with increasing aortic diameter and it often results in high mortality (2, 3). Despite the high prevalence of this disease, there is no effective pharmacological therapy that reduces the diameter of AAA. Therefore, current therapy for AAA is restricted to surgical or endovascular repair.

Pathological features of AAA involve the degradation of extracellular matrix, such as elastin and collagen, the depletion of smooth muscle cells, and the infiltration of lymphocytes and macrophages (4). Inflammatory cell infiltration within the aortic wall plays an important role during the process of AAA formation by releasing cytokines and proteases (5–8). In addition, macrophage infiltration and proinflammatory cytokine expression in periaortic adipose tissue surrounding abdominal aortas enhances AAA formation in mice (9).

Invariant natural killer T (iNKT) cells are innate-like T lymphocyte population characterized by co-expressing natural killer lineage receptors and T cell receptors (TCR) with the invariant α-chain (Vα14-Jα18 in mice and Vα24-Jα18 in humans). iNKT cells recognize glycolipid antigens and rapidly and robustly produce a mixture of T helper type (Th) 1 and Th2 cytokines such as interferon (IFN)-γ and interleukin (IL)-4 that shape subsequent immune responses on activation (10). iNKT cells can regulate the Th1/Th2 balance and, by this means, can function as immunomodulatory cells in the various pathological processes including atherogenesis and metabolic disorders in obesity (11–13).

Previous studies demonstrated that iNKT cells were infiltrated into the aortic wall of human AAA (14). This findings suggest that iNKT cells can impact on the development of AAA. However, the effect of iNKT cells in AAA is largely unexplored. In the present study, we aimed to elucidate whether iNKT cell activation impact on the development of AAA.

## Materials and Methods

### Experimental Mice

Male 8-week-old leptin deficient *ob/ob* mice on C57BL/6 background (Charles River Japan, Yokohama, Japan) were obtained and weaned onto a standard diet. At 9 weeks of age, angiotensin II (AngII; 1,000 ng/kg/min; Sigma-Aldrich, St Louis, MO, USA) or phosphate-buffered saline (PBS) was administered subcutaneously by osmotic minipumps (model 2004; Alzet, Cupertino, CA, USA). Mice were divided into 2 groups according to the intraperitoneal injection of α-galactosylceramide (αGC; 0.1 μg/g body weight; Funakoshi Co., Ltd., Tokyo, Japan), the activator of iNKT cells, or PBS twice a week for 1 week in Experiment 2 and Experiment 3, or 4 weeks in Experiment 1 and Experiment 3 (Supplementary Figure 1). The concentration of αGC was chosen based on the previous study of its efficacy (9). At the end of the experiments, the mice were anesthetized by overdose of pentobarbital sodium (100 mg/kg) and euthanized by collection of blood from right ventricle. The animal care and procedures for the experiments (13-0073) were approved by the Committee of Hokkaido University Faculty of Medicine and Graduate School of Medicine on Animal Experimentation and conformed the Guide for the Care and Use of Laboratory Animals published by the US National Institutes of Health.

#### Experiment 1: The Effects of αGC Administration on AngII-Mediated AAA

The experiment was performed in the following 4 groups of mice; PBS-PBS (*n* = 10), PBS-αGC (*n* = 5), AngII-PBS (*n* = 6), and AngII-αGC (*n* = 12) (Supplementary Figure 1). Four weeks after administration of PBS or AngII, systolic blood pressure was measured under conscious state by using a tail cuff system (BP 98A; Softron, Tokyo, Japan). Afterwards, the mice were euthanized and abdominal aortic aortae were dissected for the histomorphometric analysis.

#### Experiment 2: The Effects of αGC Administration on iNKT Cell Infiltration to Aortic Tissues

To quantify iNKT cell infiltration to aortic tissues, the experiment was performed in the following 4 groups of mice; PBS-PBS (*n* = 3), PBS-αGC (*n* = 4), AngII-PBS (*n* = 4), and AngII-αGC (*n* = 3) (Supplementary Figure 1). One week after administration of PBS or AngII, the mice were euthanized and abdominal aortic tissues were dissected for the flowcytometric analysis.

#### Experiment 3: The Effects of αGC Administration on Gene Expression of Aortic Tissues

To quantify gene expression of aortic tissues, the experiment was performed in the following 3 groups of mice; PBS-PBS (*n* = 16), AngII-PBS (*n* = 12), and AngII-αGC (*n* = 13) (Supplementary Figure 1). One or 4 weeks after administration of PBS or AngII, the mice were euthanized and abdominal aortic tissues were dissected for the quantitative realtime PCR analysis.

### Blood Chemistry

After fasting for 16 h, blood was collected from right ventricle to obtain plasma at the time of euthanasia, which was stored at −80°C. Plasma levels of non-HDL cholesterol and triglyceride were determined enzymatically by using assay kits (Wako Pure Chemical Industries, Osaka, Japan) as recommended by the manufacturer. The samples were assayed in duplicate.

### Histomorphometric Analysis

Mice were perfused via left ventricular puncture with 10% neutralized buffered formaldehyde under physiological pressure. The maximum diameter of abdominal aorta was measured from the image captured by a digital camera (CAMEDIA C-7070; Olympus, Tokyo, Japan) attached to a dissecting microscope. The presence of an aneurysm was defined as >50% increase in maximum diameter of abdominal aorta. The segments of abdominal aorta with maximum diameter were embedded in paraffin. Serial sections of 5-μm thickness were stained with elastica-van Gieson and captured with a BZ-8000 microscope (Keyence, Osaka, Japan).

### Immunohistochemistry

To quantify the infiltration of macrophages and T lymphocytes in the aortic tissues, three different sections of abdominal aorta with maximum diameter were stained with antibody (Ab) against mouse F4/80 [rat anti-mouse F4/80 monoclonal Ab (mAb); Abcam, Cambridge, UK], a specific maker for mature macrophages, or CD3 (rabbit anti-human CD3 polyclonal Ab; Dako, Carpentaria, CA, USA), a specific marker for T lymphocytes, followed by counter-staining with hematoxylin. Three different fields from each section were randomly selected from each section. Within each field, the F4/80- or CD3-positive area was quantified using image analysis software (Image J version1.47, National Institutes of Health, Bethesda, MD) and the macrophage or T lymphocyte infiltration ratio was then expressed as a percentage based upon the ratio of sum of three F4/80- or CD3-positive areas divided by the sum of the three aortic tissue area.

### Flow Cytometry

Aortic tissues including descending thoracic aorta and abdominal aorta were digested with collagenase type I (450 U/mL; Sigma-Aldrich, St Louis, MO, USA), collagenase type XI (125 U/mL; Sigma-Aldrich), hyaluronidase type I (60 U/mL; Sigma-Aldrich), and DNase I (60 U/mL; Sigma-Aldrich) in PBS containing 20 mmol/L HEPES at 37°C for 1 h (15). A cell suspension was obtained by mashing the aorta through a 70 μm cell strainer. Cells were incubated with 2.4G2 mAb (anti-FcγRII/III) to block non-specific binding of Ab, and were stained with a combination of V450 conjugated anti-CD45 mAb (BD Biosciences, San Jose, CA, USA), fluorescein isothiocyanate-conjugated anti-TCRβ chain mAb (BD Biosciences), and R-Phycoerythrin-conjugated murine CD1d tetramer pre-loaded with αGC (ProImmune Ltd., Oxford, UK). Stained cells were acquired with FACS CantoTM II flow cytometer (BD Biosciences) and analyzed with FlowJo (TreeStar Inc., Ashland, OR, USA) software. 7-Amino-Actinomycin D (BD Biosciences)-positive cells were electronically gated out from analysis. iNKT cell population was defined as CD45-, αGC-CD1d-Tetramer-, and TCRβ-positive cell.

### Quantitative Reverse Transcriptase PCR

Total RNA was extracted from whole aortic tissues with RNeasy micro kit (QIAGEN, Tokyo, Japan) according to the manufacture's protocol. cDNA was synthesized with the high capacity cDNA reverse transcription kit (Applied Biosystems, Foster City, CA, USA). TaqMan quantitative PCR was performed with the 7300 real-time PCR system (Applied Biosystems) to amplify samples for major histocompatibility complex (MHC) class II (a marker for macrophage activation), CD11c (a marker for M1 macrophages), regulated on activation, normal T cell expressed and secreted (RANTES), monocyte chemotactic protein-1 (MCP-1), arginase-1, resistin-like molecule alpha (RELMa), Chitinase 3-like protein-3 (Chi3l3, also termed as Ym1), C-type mannose receptor 1 (MRC1, also termed as CD206) (markers for M2 macrophages), matrix metalloproteinase (MMP)-2, MMP-9, IFN-γ, tumor necrosis factor (TNF)-α, IL-4, and IL-10. These transcripts were normalized to GAPDH. All primers were purchased from Applied Biosystems.

### Statistical Analysis

Data were expressed as the means ± S.D. Statistical analysis was performed using one-way or two-way ANOVA with the Tukey *post-hoc* test (GraphPad Prism 9.02; GraphPad Software, San Diego, CA, USA). A *p*-value < 0.05 was considered statistically significant.

## Results

### αGC Ameliorated the Development of AngII-Mediated AAA

AngII significantly increased systolic blood pressure and plasma non-HDL cholesterol compared to PBS-treated mice (Table 1). αGC did not affect body weight, systolic blood pressure, and plasma non-HDL cholesterol and triglyceride.

**Table 1:** Animal characteristics.

	**PBS-PBS**	**PBS-αGC**	**AngII-PBS**	**AngII-αGC**
	**(*n* = 10)**	**(*n* = 5)**	**(*n* = 6)**	**(*n* = 12)**
Body weight, g	46.7 ± 2.5	48.4 ± 1.4	46.4 ± 2.0	44.4 ± 4.4
Systolic blood pressure, mmHg	112 ± 9	107 ± 7	154 ± 13^*^	161 ± 20^*^
**Blood chemistry**				
Non-HDL cholesterol, mg/dL	93 ± 26	94 ± 19	144 ± 19^*^	124 ± 31^*^
Triglyceride, mg/dL	56 ± 13	42 ± 12	81 ± 36	77 ± 18

*PBS, phosphate-buffered saline; αGC, α-galactosylceramide; AngII, angiotensin II*.

* *P < 0.01 vs. PBS-PBS. All data are means ± S.D*.

**Figure 1:**
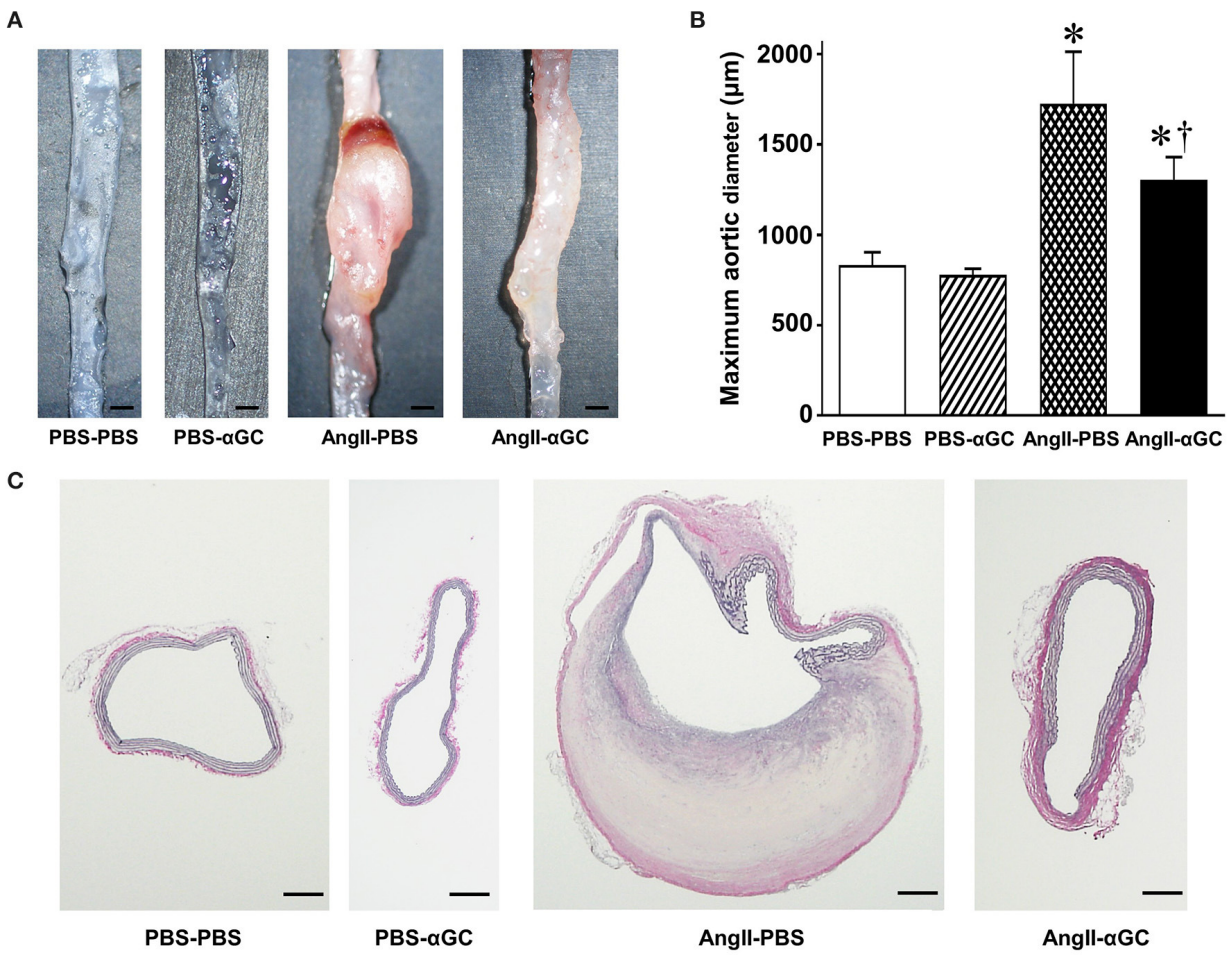
Activation of invariant natural killer T (iNKT) cells by α-galactosylceramide (αGC) ameliorated the development of angiotensin II-mediated abdominal aortic aneurysm in mice. **(A)** Representative pictures of abdominal aorta from 4 groups of PBS-PBS, PBS-αGC, AngII-PBS, and AngII-αGC mice 4 weeks after administration of PBS or AngII. Scale bar = 500 μm. **(B)** Maximum aortic diameter from 4 groups of PBS-PBS (*n* = 10), PBS-αGC (*n* = 5), AngII-PBS (*n* = 6), and AngII-αGC (*n* = 12). **(C)** Representative photomicrographs of elastica-van Gieson's staining of cross-sections at abdominal aorta from the 4 groups of mice. Scale bar = 200 μm. *P < 0.05 vs. PBS-PBS, ^†^*P* < 0.05 vs. AngII-PBS. All data are means ± S.D. PBS: phosphate- buffered saline, αGC, α-galactosylceramide; AngII, angiotensin II.

AngII induced supra-renal AAA in AngII-PBS (6 out of 6 mice) and AngII-αGC (8 out of 12 mice), while AAA lesion was not induced in PBS-PBS and PBS-αGC. The maximal abdominal aortic diameter was increased by 2.1-folds in AngII-PBS compared to PBS-PBS (*P* < 0.0001) and this increase was ameliorated by 0.72-folds in AngII-αGC compared to AngII-PBS (*P* = 0.0003; Figures 1A,B). The abdominal aortic diameter of PBS-αGC did not differ with that of PBS-PBS.

Histological analysis with Elastica van Gieson's staining demonstrated that AngII induced the localized degeneration of the medial extracellular matrix with disruption of elastic lamella and intramural hemorrhage in AngII-PBS (Figure 1C). These histological changes were attenuated in AngII-αGC. PBS-PBS or PBS-αGC exhibited no such structural changes of the aorta.

### αGC Activated iNKT Cells and Decreased Inflammatory Cell Infiltration to the Aorta

The flow cytometric analysis 1 week after the administration of PBS or AngII demonstrated that the proportion of iNKT cells to mononuclear cells was increased by 2.4- and 2.5-folds in aortic tissues from PBS-αGC and AngII-αGC compared to PBS-PBS (*P* = 0.03, each; Supplementary Figure 2). However, it did not differ between PBS-αGC and AngII-αGC.

**Figure 2:**
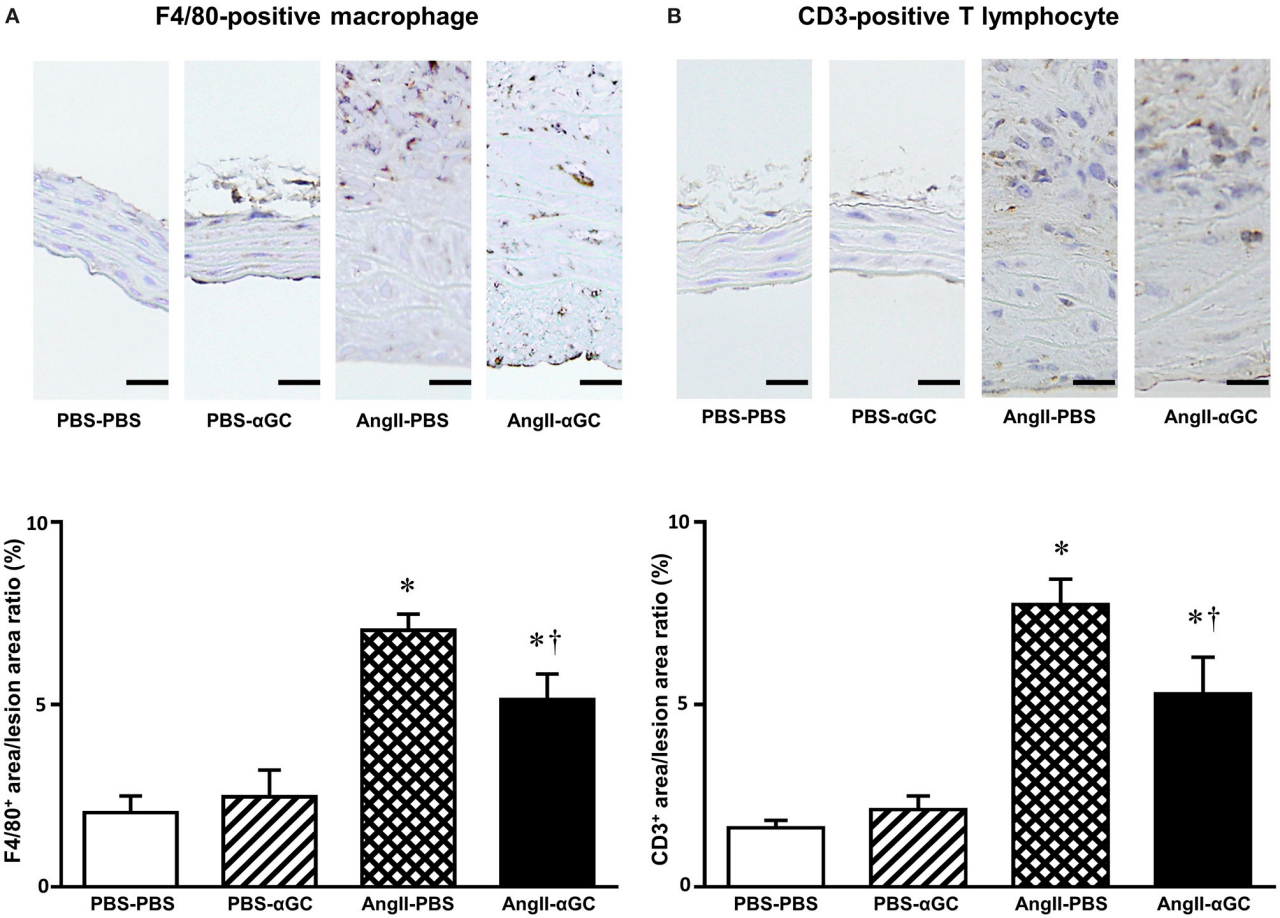
α-galactosylceramide (αGC) decreased inflammatory cell infiltration to the aorta. Representative photomicrographs of anti-F4/80 **(A)** and anti-CD3 **(B)** immunohistochemistry at suprarenal aorta from 4 groups of PBS-PBS, PBS-αGC, AngII-PBS, and AngII-αGC mice at 4 weeks after administration of PBS or AngII. Scale bar = 20 μm. Summary data of the ratio of F4/80^+^ area to the lesion area and CD3^+^ area to the lesion area from 4 groups of PBS-PBS (*n* = 10), PBS-αGC (*n* = 5), AngII-PBS (*n* = 6), and AngII-αGC (*n* = 12) mice were shown in the panels below. *P < 0.05 vs. PBS-PBS. ^†^*P* < 0.05 vs. AngII-PBS. All data are means ± S.D. PBS, phosphate-buffered saline; αGC, α-galactosylceramide; AngII, angiotensin II.

To examine the cellular components of aortic tissues 4 weeks after administration of PBS or AngII, tissue sections with maximum diameter were analyzed with immunohistochemistry. F4/80-positive macrophages were enhanced by 3.4-folds in the media and the adventitia from AngII-PBS compared to PBS-PBS (*P* = 0.0002) and this increase was diminished by 0.70-folds in AngII-αGC compared to AngII-PBS (*P* = 0.04; Figure 2A). In parallel to the macrophage infiltration, CD3-positive lymphocytes were infiltrated to the lesion by 4.8-folds in AngII-PBS compared to PBS-PBS (*P* = 0.0002), and this increase was also diminished by 0.68-folds in AngII-αGC compared to AngII-PBS (*P* = 0.0498; Figure 2B).

### αGC Induced M2-Polarization of Macrophages in the Aorta

**Figure 3:**
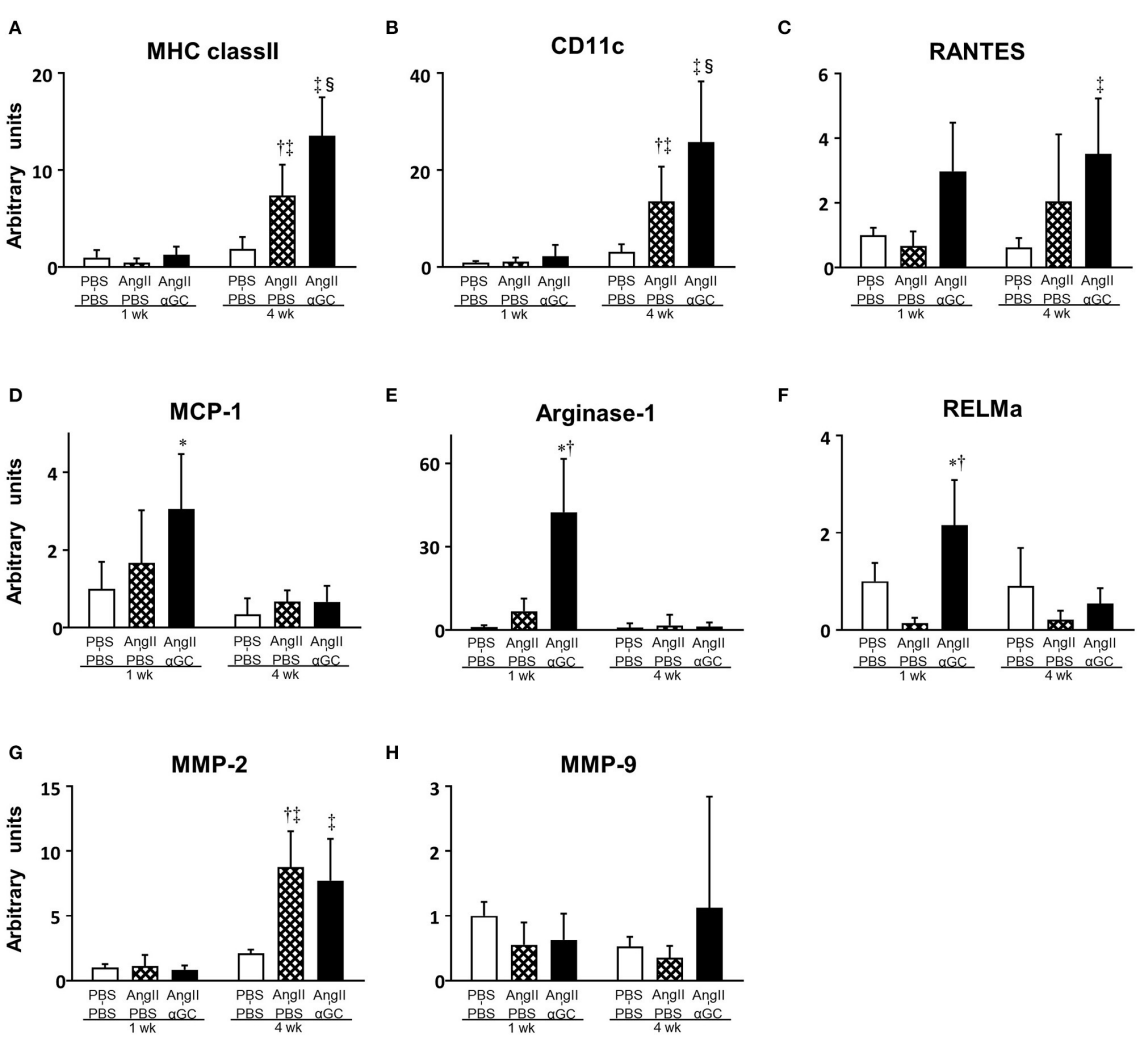
α-galactosylceramide (αGC) induced M2-polarization of macrophages in the aorta. Quantitative analyses of mRNA expression of MHC class II **(A)**, CD11c **(B)**, RANTES **(C)**, MCP-1 **(D)**, Arginase-1 **(E)**, RELMa **(F)**, MMP-2 **(G)**, and MMP-9 **(H)** in aortic tissues from 3 groups of PBS-PBS, AngII-PBS, and AngII-αGC mice 1 or 4 weeks after administration of PBS or AngII (*n* = 6–10 per group). Data are means ± S.D. *P < 0.05 vs. PBS-PBS (1 week), ^†^*P* < 0.05 vs. AngII-PBS (1 week), ^‡^*P* < 0.05 vs. PBS-PBS (4 week), ^§^*P* < 0.05 vs. AngII-PBS (4 week). MHC, major histocompatibility complex; RANTES, regulated on activation, normal T cell expressed and secreted; MCP-1, monocyte chemotactic protein-1; RELMa, resistin-like molecule alpha; MMP, matrix metalloproteinase; PBS, phosphate-buffered saline; αGC, α-galactosylceramide; AngII, angiotensin II.

MHC class II (macrophage marker), CD11c (M1 macrophage marker), and RANTES (T lymphocyte marker) gene expression was upregulated by 3.9-, 4.3-, and 3.3-folds in AngII-PBS compared to PBS-PBS (*P* = 0.0002, *P* = 0.017, and *P* = 0.23, respectively) and this increase was further enhanced by 1.8-, 1.9-, and 1.7-folds in AngII-αGC compared to AngII-PBS at 4 weeks (*P* = 0.002, *P* = 0.008, and *P* = 0.28, respectively), while such changes were not observed at 1 week (Figures 3A–C). MCP-1 gene expression was increased by 3.1-folds in AngII-αGC compared to PBS-PBS at 1 week (*P* = 0.001; Figure 3D). Arginase-1 and RELMa (M2 macrophage markers) gene expression did not change in AngII-PBS compared to PBS-PBS, however, it was increased by 6.3- and 15.7-folds in AngII-αGC compared to AngII-PBS at 1 week (*P* < 0.0001, each; Figures 3E,F). Additionally, Ym1 and CD206 (M2 macrophage markers) gene expression did not change in AngII-PBS compared to PBS-PBS, however, they were increased by 2.9- and 2.4-folds in AngII-αGC compared to AngII-PBS at 1 week (*P* = 0.047 and *P* = 0.0002, respectively) (data not shown). These changes of M2 macrophage infiltration were not observed at 4 weeks. IFN-γ, TNF-α, IL-4, and IL-10 gene expression levels were below the detection sensitivity (data not shown). MMP-2 gene expression did not change at 1 week and increased by 4.2- and 3.7-folds in AngII-PBS and AngII-αGC compared to PBS-PBS at 4 weeks (*P* < 0.0001, each; Figure 3G). MMP-9 gene expression was not altered in any groups (Figure 3H).

## Discussion and Conclusions

The present study demonstrated that AngII infusion in obese *ob/ob* mice developed AAA formation with the accumulating of macrophages and T lymphocytes within the vascular tissues, and local degeneration of the medial extracellular matrix. The activation of iNKT cells by αGC attenuated the development of AngII-mediated AAA formation via inhibiting the infiltration of macrophages and T lymphocytes within the vascular tissues and inducing M2 macrophage infiltration at early phase.

Apolipoprotein-E (ApoE) deficient mice have been used with subcutaneous AngII infusion by osmotic minipumps to induce AAA (16). This mouse model recapitulates some features of AAA formation including medial degeneration and inflammation in human. AngII is known to induce vascular inflammation via the production of reactive oxygen species (ROS), inflammatory cytokines, and adhesion molecules (17). Therefore, AngII-mediated AAA was associated with vascular inflammation as well as medial degradation and subsequent vascular hematoma in ApoE deficient mice (18). However, ApoE, a multifactorial component of plasma lipoproteins, has been reported to bind and traffic antigens as the molecular chaperone for iNKT cell activation (19). Police et al. demonstrated that obesity increases macrophage infiltration and cytokine expression in periaortic adipose tissue and enhances AngII-mediated AAA formation in *ob/ob* mice in the presence of ApoE (9). Therefore, to elucidate the impact of iNKT cell activartion on the development of AAA, we administered AngII to obese *ob/ob* mice and demonstrated that aneurysmal aorta was surrounded by a dozen of periaortic adipose tissues, consistent with human AAA. They had local degeneration of the medial extracellular matrix with disruption of elastic lamella and intramural hemorrhage, and developed aortic aneurysm formation in association with the infiltration of macrophages and lymphocytes.

The infiltration and activation of macrophages as well as lymphocytes within aorta play a pivotal role in the development of AAA. Boytard et al. demonstrated that both M1 and M2 macrophages are differentially-localized in human AAA: M1 macrophages were predominant in the adventitia layer, where the aortic wall is more degraded, and M2 macrophages were predominant in the luminal portion among the intraluminal thrombus (20). AngII induces oxidative stress in the vascular tissue during the early course of AAA formation and ROS lead to the activation of M1 macrophages, which produce more ROS, resulting in a vicious cycle to exacerbate AAA formation (21). Moreover, Rateri et al. demonstrated that M2 macrophages were found to accumulate at rupture sites of AAA in ApoE deficient mice (22). M2 macrophages play a pivotal role in resolving inflammation during tissue remodeling process (20), suggesting that M2 macrophages attenuate chronic inflammation in the vascular tissues from AngII-αGC mice.

Recently, M2 subset of macrophages can be further divided into several subsets that play different roles (23). The M2a macrophages, which highly express CD206 in response to IL-4 and IL-13 signaling, are involved in tissue remodeling. The M2b macrophages, which are induced by Toll-like receptor agonists, play a role in immunoregulation. The M2c macrophages, which express arginase-1 in response to IL-10, are responsible for removing apoptotic cells. The present study suggested that iNKT cell activation by αGC skeweed the polarization into M2c macrophages at early phase. Since macrophages are characterized by great plasticity which dynamically changes their phenotype in response to various signals, further investigation should be needed to illustrate the underlying mechanisms.

iNKT cells are a unique subset of T lymphocytes that recognize glycolipid antigens (10). They can rapidly and robustly produce a mixture of Th1 and Th2 cytokines on activation in shaping subsequent immune responses. Indeed, in the preset study, the flow cytometric analysis revealed that αGC administration induced iNKT cell infiltration into aortic tissues 1 week after the administration. By contrast, AngII administration did not affect the iNKT cell infiltration in the tissues, suggesting that iNKT cells impact on the AAA formation solely as a modifier. The present study did not elucidate whether iNKT cells are involved in the development of AAA, activation of iNKT cells can be a novel therapeutic target against the AAA formation via inducing anti-inflammatory M2 polarized state. Several pharmacological approaches have been postulated to regulate immunological responses, however they have not been applied in the clinical settings (24, 25). αGC is a potent specific activator of iNKT cells and clinical trials using αGC for heart failure or cancer patients have shown favorable effects for clinical outcomes as well as the safety and tolerability of αGC (26). These studies suggested that the bioactive lipid αGC could be a novel therapeutic agent for the AAA patients.

There are several limitations to be acknowledged in the present study. First, Ait-Oufella et al. reported that IL-10 was involved in the amelioration of AngII-induced AAA formation in mice (27). We reported that activation of iNKT cells by αGC induced Th2 response in the mouse model of post-infarct heart failure and myocardial ischemic/reperfusion injury (28, 29). In both models, the upregulation of IL-10 by iNKT cell activation play a crucial role in the amelioration of myocardial remodeling. However, the present study could not demonstrate the role of IL-10 in the beneficial effects of αGC in AAA formation. Second, the infiltration of F4/80-positive macrophages and CD3-positive lymphocytes were significantly diminished in AngII-αGC compared to AngII-PBS by immunohistochemistry (Figure 2). On the contrary, gene expression of MHC class II, CD11c, and RANTES was upregulated in AngII-αGC compared to AngII-PBS by quantitative reverse transcriptase-PCR (Figure 3). These discrepant results might be due to the difference between surface protein and gene expression. αGC induces FasL on activated iNKT cells and apoptosis of neighboring cells such as macrophages and T lymphocytes, suggesting that αGC increases the turnover of these cells (30).

In conclusions, the activation of iNKT cells by αGC plays a protective role against the development of AAA through the enhanced expression of M2 macrophages. Therapies designed to activate iNKT cells might be beneficial to prevent from developing AAA.

## Data Availability Statement

The raw data supporting the conclusions of this article will be made available by the authors, without undue reservation.

## Ethics Statement

The animal study was reviewed and approved by Committee of Hokkaido University Faculty of Medicine and Graduate School of Medicine on Animal Experimentation.

## Author Contributions

AS designed experiments, performed experiments, analyzed data, and wrote the manuscript. NI conceived, designed experiments, and wrote the manuscript. ST, MN, and TH performed experiments, analyzed data, and contributed to discussions. KI and HT designed experiments, contributed to discussions, reviewed, and edited the manuscript. All authors have read and approved the manuscript.

## Conflict of Interest

The authors declare that the research was conducted in the absence of any commercial or financial relationships that could be construed as a potential conflict of interest.

## Supplementary Material

Supplementary Figure 1 Schematic design of the study. Mice were administered AngII or PBS by osmotic minipumps at 9 weeks of age and further divided into 2 groups according to the injection of αGC or PBS twice a week for 1 or 4 weeks. Treatments performed on mice were represented by downward black arrows and the day of sampling was represented by an upward blue arrow in Experiment 1 **(A)**, Experiment 2 **(B)**, and Experiment 3 **(C)**. AngII, angiotensin II; PBS, phosphate-buffered saline; αGC, α-galactosylceramide.

Supplementary Figure 2 α-galactosylceramide (αGC) activated invariant natural killer T (iNKT) cells in the aorta. **(A)** Representative flow cytometric analyses of aortic mononuclear cell suspensions from 4 groups of PBS-PBS, PBS-αGC, AngII-PBS, and AngII-αGC mice 1 week after administration of PBS or AngII. **(B)** iNKT cells/lymphocytes ratio from 4 groups of PBS-PBS (*n* = 3), PBS-αGC (*n* = 4), AngII-PBS (*n* = 4), and AngII-αGC (*n* = 3). ^*^*P* < 0.05 vs. PBS-PBS, ^†^*P* < 0.05 vs. AngII-PBS. All data are means ± S.D. PBS, phosphate-buffered saline; αGC, α-galactosylceramide; AngII, angiotensin II; iNKT, invariant natural killer T; TCRβ, T cell receptor β chain.

Supplementary Table 1 Non-standard Abbreviations and Acronyms.
