# Supplementary figures and images for: Activation of Invariant Natural Killer T Cells by α-Galactosylceramide Attenuates the Development of Angiotensin II-Mediated Abdominal Aortic Aneurysm in Obese ob/ob Mice
Source: Front Cardiovasc Med. 2021 May 10;8:659418. doi: 10.3389/fcvm.2021.659418 (PMC8141584; doi:10.3389/fcvm.2021.659418)

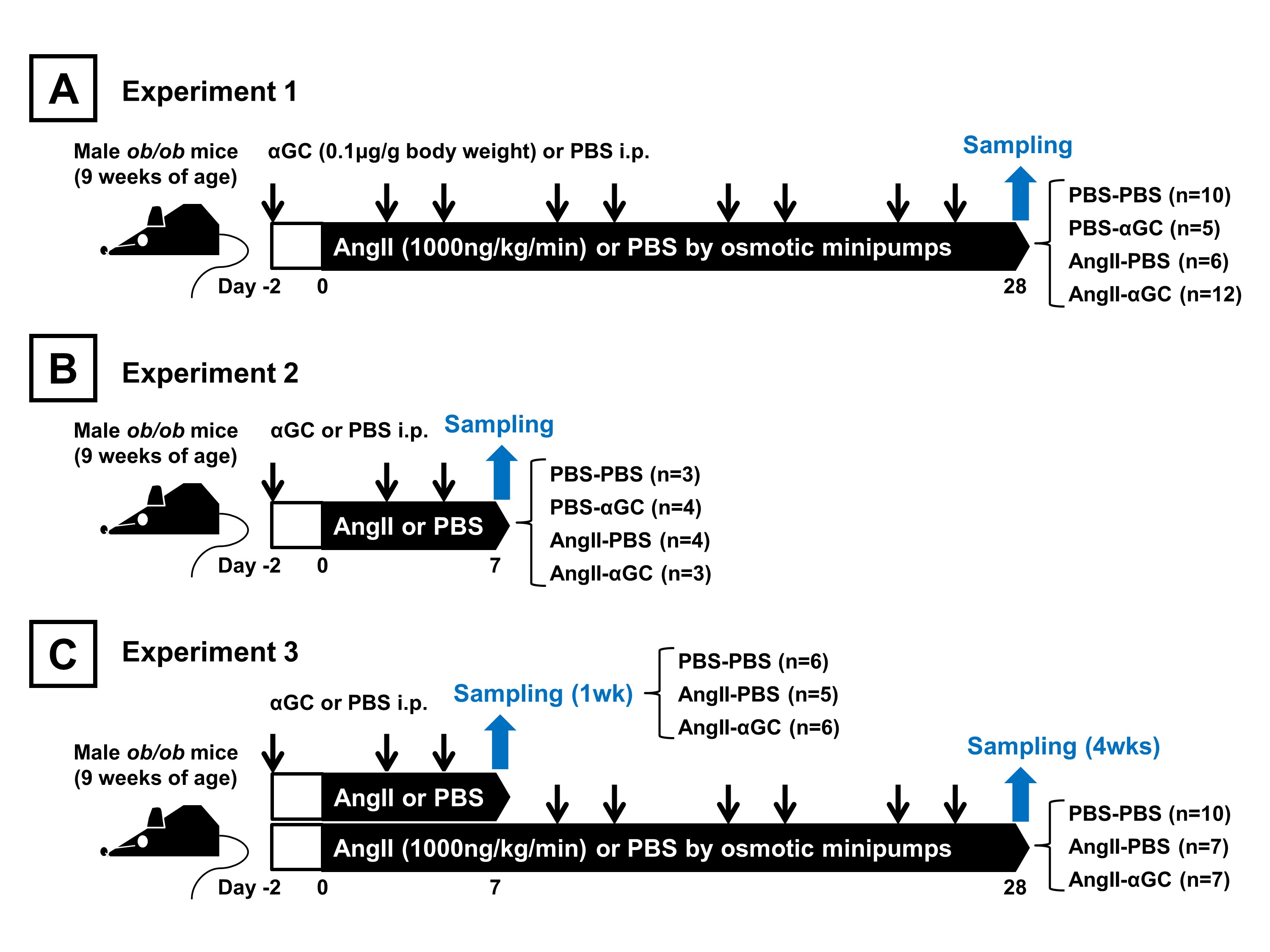

Supplement: Supplementary Figure 1 — Schematic design of the study. Mice were administered AngII or PBS by osmotic minipumps at 9 weeks of age and further divided into 2 groups according to the injection of αGC or PBS twice a week for 1 or 4 weeks. Treatments performed on mice were represented by downward black arrows and the day of sampling was represented by an upward blue arrow in Experiment 1 (A), Experiment 2 (B), and Experiment 3 (C). AngII, angiotensin II; PBS, phosphate-buffered saline; αGC, α-galactosylceramide. [file Image_1.JPEG]

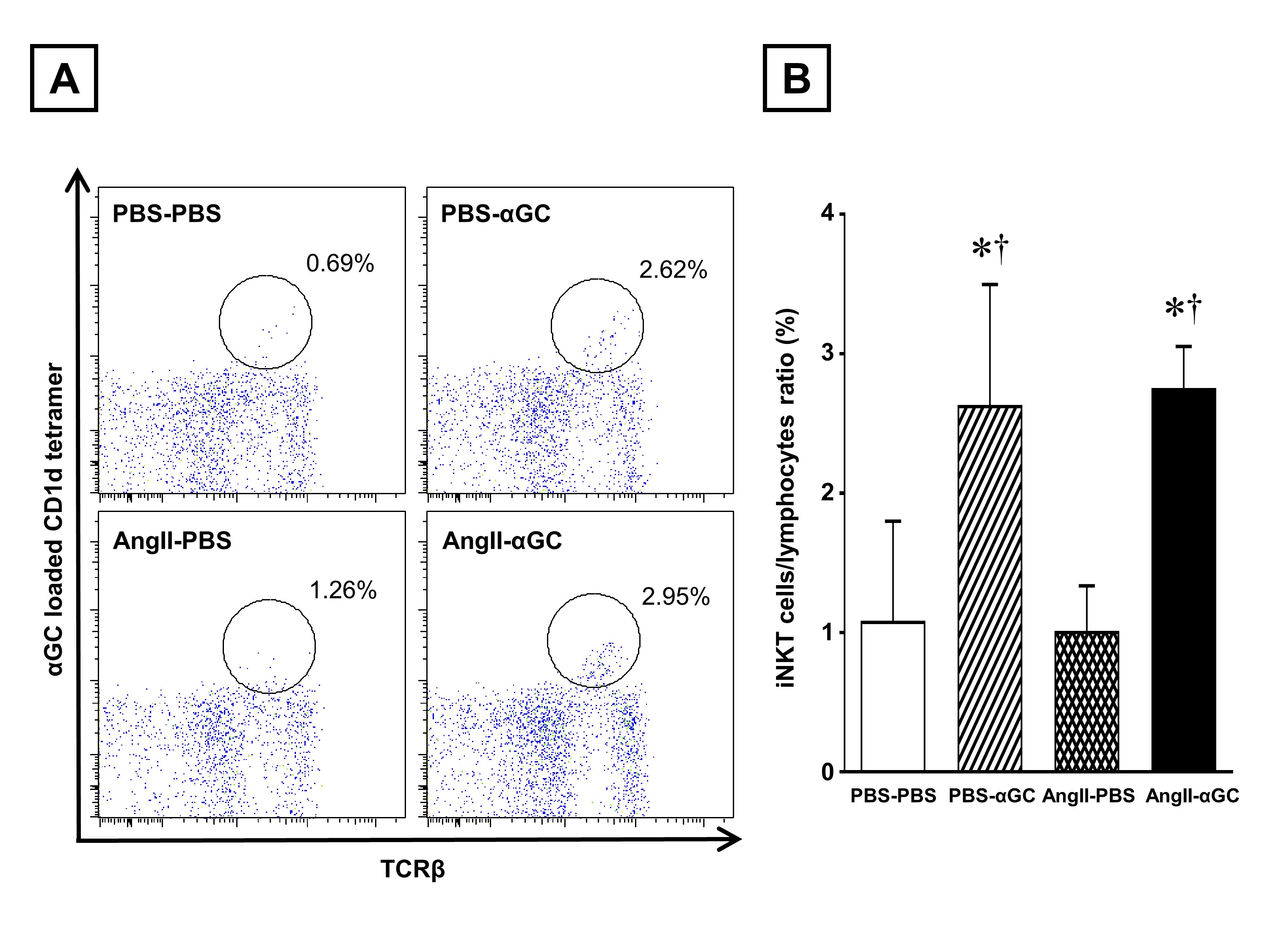

Supplement: Supplementary Figure 2 — α-galactosylceramide (αGC) activated invariant natural killer T (iNKT) cells in the aorta. (A) Representative flow cytometric analyses of aortic mononuclear cell suspensions from 4 groups of PBS-PBS, PBS-αGC, AngII-PBS, and AngII-αGC mice 1 week after administration of PBS or AngII. (B) iNKT cells/lymphocytes ratio from 4 groups of PBS-PBS (n = 3), PBS-αGC (n = 4), AngII-PBS (n = 4), and AngII-αGC (n = 3). *P < 0.05 vs. PBS-PBS, †P < 0.05 vs. AngII-PBS. All data are means ± S.D. PBS, phosphate-buffered saline; αGC, α-galactosylceramide; AngII, angiotensin II; iNKT, invariant natural killer T; TCRβ, T cell receptor β chain. [file Image_2.JPEG]
