# Supplementary material for: Activation of Invariant Natural Killer T Cells by α-Galactosylceramide Attenuates the Development of Angiotensin II-Mediated Abdominal Aortic Aneurysm in Obese ob/ob Mice
Source: Front Cardiovasc Med. 2021 May 10;8:659418. doi: 10.3389/fcvm.2021.659418 (PMC8141584; doi:10.3389/fcvm.2021.659418)
Supplement: Supplementary Table 1 — Non-standard Abbreviations and Acronyms. [file Table_1.pdf]

Supplementary Table 1

Non-standard Abbreviations and Acronyms	
AAA	abdominal aortic aneurysm
αGC	α-galactosylceramide
AngII	angiotensin II
ApoE	apolipoprotein-E
Chi3l3	chitinase 3-like protein-3
IFN	interferon
IL	interleukin
iNKT	invariant natural killer T
mAb	monoclonal antibody
MCP-1	monocyte chemotactic protein-1
MHC	major histocompatibility complex
MMP	matrix metalloproteinase
MRC	c-type mannose receptor 1
PBS	phosphate-buffered saline
RANTES	regulated on activation, normal T cell expressed and secreted
RELMa	resistin-like molecule alpha
ROS	reactive oxygen species
TCRβ	T cell receptor β chain
Th	T helper type
TNF	tumor necrosis factor
